# Activation of the Mesencephalic Trigeminal Nucleus Contributes to Masseter Hyperactivity Induced by Chronic Restraint Stress

**DOI:** 10.3389/fncel.2022.841133

**Published:** 2022-04-11

**Authors:** Ya-Juan Zhao, Yang Liu, Jian Wang, Qiang Li, Zhou-Ming Zhang, Teng Tu, Rong Lei, Min Zhang, Yong-Jin Chen

**Affiliations:** ^1^State Key Laboratory of Military Stomatology & National Clinical Research Center for Oral Diseases & Shaanxi International Joint Research Center for Oral Diseases, Department of General Dentistry and Emergency, School of Stomatology, Fourth Military Medical University, Xi’an, China; ^2^Department of Cardiothoracic Surgery, General Hospital of Western Theater Command, Chengdu, China

**Keywords:** chronic restraint stress, masseter muscle, mesencephalic trigeminal nucleus, trigeminal motor nucleus, vesicular glutamate transporter-1

## Abstract

Psychological stress is commonly accepted to be closely associated with masticatory muscle disorder, which is the main symptom of temporomandibular disorder (TMD). Previous studies have confirmed that exposure to stress may cause masticatory muscle hyperactivity. However, the central mechanism underlying this process remains unclear. The mesencephalic trigeminal nucleus (Vme), which resides in the brainstem, is the primary afferent center for masticatory proprioception and plays a key role in oral–motor movements by projecting to the trigeminal motor nucleus (Vmo). Therefore, the present study was designed to examine the role of Vme neurons in masseter overactivity induced by chronic stress. We found that subjecting mice to restraint stress (6 h/day) for 14 days caused significant anxiety-like behavior, obvious masseter overactivity, and markedly enhanced electrophysiological excitability of Vme neurons. By using anterograde tract tracing combined with immunofluorescence staining methods, we observed vesicular glutamate transporter 1 (VGLUT1)-positive glutamatergic projections from the Vme to the Vmo. Moreover, chronic restraint stress (CRS) elevated the expression of VGLUT1 and choline acetyltransferase (ChAT) in Vmo. Furthermore, administration of VGLUT1-targeted short hairpin RNA (shRNA) into the bilateral Vme significantly suppressed the enhanced overexcitability of Vme neurons, downregulated the overexpression of VGLUT1 and ChAT in the Vmo, and attenuated the elevated overactivity of the masseter caused by CRS. Taken together, we showed that CRS can excite neurons in the Vme, enhancing glutamatergic excitatory projections from the Vme to the Vmo and resulting in masseter muscle overactivity. These findings provide us with a novel central mechanism underlying the correlation between psychological factors and TMD.

## Introduction

Masticatory muscle disorder consists of a series of conditions, including acute/chronic orofacial pain, masticatory muscle hyperactivity comprising diurnal or nocturnal tooth clenching and grinding (bruxism), and other related symptoms of masticatory muscle dysfunction (Benoliel et al., 2011). The symptoms of masticatory muscle disorder have been reported to be the most common complaints of patients with temporomandibular disorder (TMD), which is a collective term encompassing pain and/or dysfunction of the masticatory musculature and the temporomandibular joints (Costa et al., 2018; Osiewicz et al., 2018). The orofacial symptoms caused by TMD are usually chronic and refractory, affecting oral health-related quality of life (Dahlström and Carlsson, 2010; Oghli et al., 2020).

In general, it is well-documented that psychological stress is one of the major risk factors for the occurrence and development of TMD (Fillingim et al., 2013; Slade et al., 2016). Previous studies suggested that patients with TMD tend to be more anxious and/or depressed than asymptomatic control subjects (Gameiro et al., 2006; Kindler et al., 2012; Al-Khotani et al., 2016; Simoen et al., 2020). Regarding masticatory muscle disorder, several clinical studies have confirmed that people exposed to more stress stimuli are more likely to develop a state of masticatory muscle hyperactivity or allodynia (Owczarek et al., 2020; Yap and Natu, 2020; Rofaeel et al., 2021). Consistent with these findings, data from animals under experimental stress showed that the masseter muscle significantly exhibited bruxism-like activity and metabolic dysfunction (Rosales et al., 2002; Song et al., 2014; Lin et al., 2019). Moreover, masticatory muscle overactivity and fatigue are assumed to be the main causes of TMD (Nishioka and Montgomery, 1988; Liu et al., 2019). Therefore, it must be determined whether certain central neural pathways through which stress can induce masticatory muscle overactivity exist in the brain.

The mesencephalic trigeminal nucleus (Vme) is a pair of nuclei located in the triangle between the locus coeruleus and the medial parabrachial nucleus along the entire midbrain of the brainstem (Lipovsek et al., 2017). Most Vme neurons convey the proprioceptive information of masticatory muscles and send central branches into the trigeminal motor nucleus (Vmo), which innervates the movement of masticatory muscles (Lazarov, 2002, 2007). Thus, Vme neurons play a pivotal role in oral–motor circuits and the regulation of masticatory muscle movement rhythms (Lazarov, 2007; Zhang et al., 2012; Lipovsek et al., 2017). Vme efferent neurons have been demonstrated to be mostly glutamatergic and they mostly express vesicular glutamate transporter 1 (VGLUT1) in the presynaptic terminals projecting to Vmo (Pang et al., 2006; Park et al., 2018). As the Vme contains cell bodies of primary afferent sensory neurons while being distinctively situated in the brain instead of the peripheral area, it is described as a displaced sensory ganglion (Lipovsek et al., 2017). Hence, the activities of Vme neurons may be more susceptible to other brain areas (Lazarov, 2007). However, in psychologically stressed conditions, whether Vme neurons undergo changes in excitability and thus influence masticatory muscle overactivity remains unclear.

In light of these previous studies and observations, we hypothesized that Vme neuron excitability might be enhanced by psychological stress, thereby leading to masticatory muscle hyperfunction by increasing glutamatergic projections to the Vmo. A mouse experimental stress model was established by chronic restraint. Among the masticatory muscles, the masseter is the strongest jaw elevating muscle (Almukhtar and Fabi, 2019); the majority of studies about the relationship between masticatory muscle disorder and TMD indicated a strong correlation between the masseter abnormity and TMD symptoms, such as bruxism and muscle pain (Nitecka-Buchta et al., 2019; Shah et al., 2019; Kitagawa et al., 2021). So in the present study, we selected the masseter as the representative masticatory muscle for examination. Vme neuron excitability was measured with electrophysiological recordings. Anterograde tract tracing was used to confirm the Vme-Vmo projection. To further verify the involvement of Vme neurons in masseter overactivity under stress conditions, microinjection of VGLUT1 short hairpin RNA (shRNA) into the bilateral Vme was conducted to decrease glutamatergic expression.

## Materials and Methods

### Animals

A total of 105 adult male C57BL/6 mice (20–25 g, 8 weeks old, obtained from the Laboratory Animal Center of the Fourth Military Medical University) were used in this study. The mice were caged in a room with controlled temperature (22 ± 1°C), and humidity (60 ± 5%) and a 12-h/12-h light/dark cycle (light on 8:00–20:00 h) and given access to food and water *ad libitum*. Animals were allowed to acclimate to the cages 1 week before the experiment. The study was carried out in strict accordance with the recommendations in the ethical guidelines for investigations of experimental pain in conscious animals (Zimmermann, 1983). All experimental procedures were approved by the Animal Use and Care Committee for Research and Education of the University. All efforts were made to avoid animal suffering throughout the experiment.

The present experiment consisted of three parts. The first part aimed to determine whether chronic restraint stress (CRS) induces anxiety-like behavior, masseter overactivity, and enhanced excitability of Vme neurons. Mice were randomly divided into a control (CON) group and a CRS group (*n* = 20). In the second part, neuronal tract tracing and immunofluorescence staining were carried out in 5 mice to confirm the neuronal pathway from the Vme to the Vmo. In the third part, glutamatergic expression was suppressed by injecting VGLUT1 shRNA into the bilateral Vme to further investigate the involvement of Vme neurons in masseter overactivity elicited by CRS. Mice were divided into the CON + scramble shRNA group, CRS + scramble shRNA group and CRS + VGLUT1 shRNA group (*n* = 20).

### Chronic Restraint Stress

To establish an animal model of psychological stress, the mice were subjected to restraint stress for 6 h/day for 14 consecutive days. The restraint procedure started at 8:00 *a.m.* every day. As previously described (Abe et al., 2017; Huo et al., 2017), CRS was carried out by placing each mouse in a well-ventilated 50-mL conical tube. The mice in the tube could rotate from a prone to supine position and back again freely but could not turn head to tail. During the restraint procedure, the mice were not allowed to eat or drink. The mice in the CON group were normally raised in their home cages.

### Behavioral Testing

The open-field (OF) test and elevated plus maze (EPM) test were conducted to reveal negative anxiety emotions as previously described (Wang et al., 2015; Segklia et al., 2019). The OF chamber (RD 1412-OF; RD1208-EP, Shanghai Mobile Datum Corporation, Shanghai, China) was a 50 cm (width) × 50 cm (length) × 45 cm (height) cover-free Plexiglas box settled in a temperature-controlled room and dimly illuminated by single fluorescent light over the chamber. The activity of mice for 5 min was monitored by an automated analysis system (Shanghai Mobile Datum Information Technology). The distance moved and the time spent in the center area were recorded as parameters for evaluating anxiety levels by off-line analysis. All animals were habituated to the testing room for 30 min before the test.

The EPM apparatus included two opposing open arms (OAs, 30 cm × 5 cm), two opposing closed arms (CAs, 30 cm × 5 cm × 25 cm), and a central area measuring 5 cm × 5 cm. The platform was 50 cm above the floor in a temperature-controlled room. For testing, mice were placed into the central square of the maze and were allowed to explore for 5 min. The number of OA and CA entries and the time spent in the OAs and CAs were recorded by an automated analysis system (Shanghai Mobile Datum Information Technology). The percentage of time spent in the OAs (the percentage of the total time) and the percentage of entries into the OAs (the percentage of the total entries) were measured to evaluate general anxiety levels. The habituation protocol for the EPM test was the same as that for the OF test.

### Western Blotting

To evaluate masseter activities, we detected the expression levels of acetylcholinesterase (AChE) and creatine kinase muscle-type (CK-MM) in masseter tissue. AChE is mainly distributed in the neuromuscular junctions of muscles (Silman and Sussman, 2008). Several studies have demonstrated that the AChE content in muscle tissues can be affected by stimulation or disuse of muscles (Pregelj et al., 2007; Bonansea et al., 2016). Therefore, AChE is believed to be a protein marker of muscle motor activation. In the muscle contraction process, creatine kinase (CK) plays a crucial role in cellular energy metabolism. CK-MM is the predominant isoform of the CK isoenzyme in skeletal muscle (Hornemann et al., 2003). CK-MM is generally used as a biochemical marker to estimate muscular cell injuries after excessive exercise (Brancaccio et al., 2007; Miranda-Vilela et al., 2012). In addition, we measured the expression level of choline acetyltransferase (ChAT) in the Vmo to assess the excitability of Vmo motor neurons, as ChAT is the key enzyme responsible for acetylcholine synthesis and usually serves as a marker for motor neurons (Salvaterra, 1987; Lee et al., 2019).

For Western blotting, the animals were deeply anesthetized with an overdose of sodium pentobarbital (60 mg/kg, *i.p.*) and rapidly sacrificed on ice. The bilateral masseter muscles and Vmo regions (4.84–5.34 mm caudal to Bregma, Paxinos and Franklin, 2001) in the brainstem were quickly dissected out and removed into centrifuge tubes placed on ice. Then, the selected tissue of each group was homogenized with ultrasonication in ice-cold radioimmunoprecipitation assay (RIPA) buffer. Homogenates were centrifuged at 4°C and 12,000 rpm for 10 min. The supernatant was collected, and the protein concentrations of the homogenate were determined using the BCA Protein Assay Kit (Pierce, Rockford, IL, United States) and denatured at 95°C for 5 min with 5 × SDS-loading buffer. The proteins of interest were separated by SDS–PAGE electrophoresis (30 μg of total protein per well) and transferred onto a polyvinylidene difluoride membrane (PVDF, Immobilon-P, Millipore, Billerica, MA, United States). The membranes were placed in a blocking solution (TBS with 0.02% Tween and 5% non-fat dry milk powder) for 2 h and incubated overnight with rabbit anti-AChE IgG (1:1000, Abcam, Cambridge, MA, United States), rabbit anti-CK-MM IgG (1:1000, Abcam, Cambridge, MA, United States), rabbit anti-ChAT IgG (1:1000, Abcam, Cambridge, MA, United States), and rabbit anti-VGLUT1 IgG (1:1000, Abcam, Cambridge, MA, United States). After washing, the membranes were incubated in peroxidase-conjugated secondary antibody (goat anti-rabbit 1:5000 goat anti-mouse 1:5000; Amersham Pharmacia Biotech, Piscataway, NJ, United States) for 1 h, and then the membranes were detected by the enhanced chemiluminescence detection method. The concentrations of β-actin, a housekeeping protein, were also measured by using mouse anti-β-actin antibody (1:2000, Sigma, St. Louis, MO, United States) as an intracontrol. The densities of protein blots were analyzed by using Image Lab Software (Bio–Rad, United States) and normalized to β-actin levels.

### Electrophysiological Recording

After deep anesthesia with an overdose of sodium pentobarbital (60 mg/kg, *i.p.*), the mice were sacrificed by cervical dislocation, and the brains were immediately removed and placed in oxygenated (95% O_2_ and 5% CO_2_) ice-cold artificial cerebrospinal fluid (ACSF) containing 124 mM NaCl, 2.5 mM KCl, 2 mM MgSO_4_ + 7H_2_O, 2 mM CaCl_2_, 1 mM NaH_2_PO_4_, 25 mM NaHCO_3_, 25 mM glucose, 1 mM ascorbate, and 3.0 mM pyruvate. Transverse slices (300 μm) containing the Vme (5.02–5.68 mm caudal to Bregma, Paxinos and Franklin, 2001) were cut on a Leica vibratome (Leica VT 1200 s, Heidelberger, Nussloch, Germany) and transferred to a room temperature-submerged recovery chamber with oxygenated ACSF and recovered for 1 h. Then, the slices were placed in a recording chamber on the stage of an Olympus microscope with infrared digital interference contrast optics for visualization of whole-cell patch-clamp recordings. In current clamp mode, the firing patterns of Vme neurons were compared by analyzing the trains of action potentials (APs) evoked by intracellular injection of 10–100-pA depolarizing currents for 400 ms. In voltage clamp mode, currents were recorded at a holding potential of −70 mV (recording pipettes were filled with solution containing 120 mM potassium gluconate, 20 mM KCl, MgCl_2_ 2 mM, 2 mM Na_2_-ATP, Na-GTP 0.5 mM, 0.5 mM EGTA, and 20 mM HEPES, pH 7.2–7.4; osmolality 300 mOsm), and spontaneous excitatory postsynaptic currents (sEPSCs) were recorded 10 min after establishing whole-cell access and the current reached a steady state. All signals were recorded using a Multi Clamp 700B Amplifier (Axon Instruments, Forster City, CA, United States) connected to a computer installed with pClAMP 10.0 software (Axon Instruments).

### Neuronal Tract Tracing and Immunofluorescence Staining

Five mice were used to investigate the neuronal pathway from the Vme to the Vmo using the anterograde tract tracing method. The mice were anesthetized with sodium pentobarbital [40 mg/kg, *i.p.* and were placed into a stereotaxic frame (RWD Life Science, Shenzhen, China)] after the pain withdrawal reflex disappeared. The right parietal bone was partially removed with a dental drill, and a glass micropipette (10–15 mm thick tip) filled with 0.05 μl 10% biotinylated dextran amine (BDA; 10000 MW, Molecular Probes, Eugene, OR, United States) in 0.9% saline connected to a microsyringe (1 μL, Hamilton) was advanced into the right Vme. Stereotaxic coordinates of the Vme were obtained from the Mouse Brain Atlas (Paxinos and Franklin, 2001). BDA injections were performed by pressure with a microinjection pump (RWD Life Science, Shenzhen, China) over a period of 4–6 min. At the end of the injection, the pipette was held in place for 10 min to ensure tracer absorption into the tissue and reduction of possible spread. Then, the incision was closed by suturing. An antibiotic (cefotiam hydrochloride, 66 mg/kg, *i.p.*) and analgesic (flurbiprofen axetil, 3.3 mg/kg, *i.p.*) were administered before allowing the mice to recover. After a survival time of 5–7 days, the mice were deeply anesthetized and perfused transcardially with 0.01 M phosphate buffered saline (PBS, pH 7.4), followed by 200 ml 0.1 M phosphate buffer (PB, pH 7.4) containing 4% paraformaldehyde. The brains were obtained and postfixed for 4 h and then transferred to 30% sucrose in 0.1 M PB for dehydration. Thirty-micrometer-thick transverse frozen sections of the brainstem were cut with a cryostat (Leica CM1800; Heidelberg, Germany) and collected in 0.01 M PBS. The sections were rinsed, blocked with 10% normal donkey serum in 0.01 M PBS for 30 min at room temperature and then used for immunofluorescent visualization of BDA (injection sites in the Vme/projecting fibers and terminals in the Vmo), VGLUT1, and Vmo neuronal somas. The sections were incubated overnight at room temperature with primary antibodies: rabbit anti-VGLUT1 (1:500, Synaptic Systems, Göettingen, Germany) and goat anti-ChAT (1:500, Millipore, Massachusetts, MA, United States). Additionally, 0.01 M PBS instead of primary antibody was used as a negative control. After being rinsed, the sections were incubated for 4 h at room temperature with a cocktail of Alexa Fluor 488-avidin (1:1000; Thermo Fisher Scientific, Inc., Waltham, MA, United States), Alexa Fluor 594-conjugated donkey anti-rabbit IgG (1:500; Thermo Fisher Scientific, Inc., Waltham, MA, United States) and Alexa Fluor 647-conjugated donkey anti-mouse IgG (1:500; Thermo Fisher Scientific, Inc., Waltham, MA, United States). Confocal images were obtained using a confocal laser microscope (FV1000; Olympus, Tokyo, Japan), and digital images were captured and processed with Fluoview 1000 (Olympus, Tokyo, Japan).

### Injection of Viral Vector

Mouse VGLUT1 shRNA-encoded adeno-associated viral (AAV) vectors containing sequences expressing enhanced green fluorescent protein [EGFP; pAAV(shRNA)-EGFP-U6 > mSlc17a7, 1.71 × 10^12^ GC/mL; Hunan Fenghui Bioscience Co., Limited, China] and corresponding scramble shRNA [pAAV(shRNA)-CMV > EGFP-U6 > Scramble, 2.05 × 1011 GC/mL; Hunan Fenghui Bioscience Co., Limited, China] were produced. The VGLUT1 shRNA vector served as RNA interference to downregulate VGLUT1 messenger RNA (mRNA) expression. In the third part of the experiment, the mice were divided into CON + scramble shRNA group, CRS + scramble shRNA group and CRS + VGLUT1 shRNA group (*n* = 20). The scramble shRNA was injected into the bilateral Vme (0.3 μl unilaterally) of mice in CON + scramble shRNA group and CRS + scramble shRNA group. The VGLUT1 shRNA was injected into the bilateral Vme (0.3 μl unilaterally) of mice in CRS + VGLUT1 shRNA group. The injections were carried out similar to that used for tracer injections. After 1 week, the mice in the CRS + scramble shRNA group and CRS + VGLUT1 shRNA group were subjected to restraint stress for 14 days. Then, OF and EPM tests, Western blotting, and electrophysiological recordings were carried out (see in Figure 4B).

**FIGURE 1:**
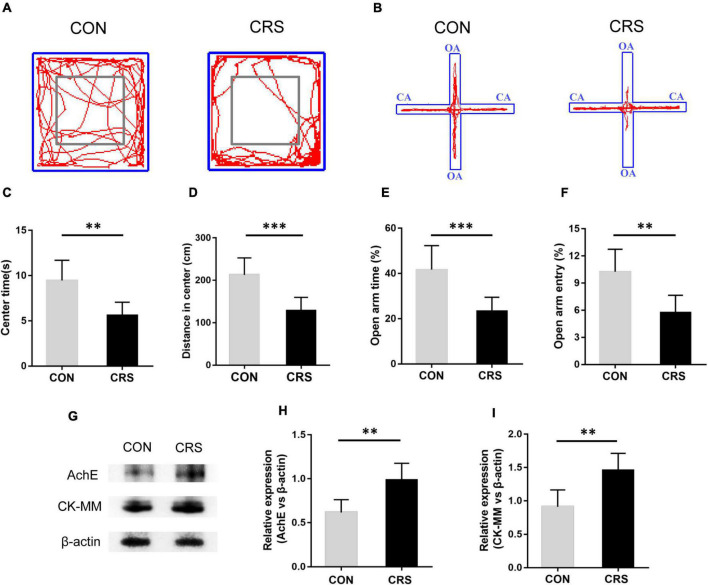
Effect of CRS on behavioral changes and masseter AChE and CK-MM expression. Representative movement trials of mice from the CON and CRS groups in the OF **(A)** and EPM **(B)** tests (*n* = 6/group). The gray square frames represent the center area in the OF. OA: open arm. CA: closed arm. In the OF test, the time spent in the center area **(C)** and the distance moved in the center area **(D)** were significantly different between the CON and CRS groups. In the EPM test, the percentage of the open-arm retention time **(E)** and open-arm entries **(F)** were significantly different between the CON and CRS groups. **(G)** Western blotting analysis displayed the protein levels of AChE and CK-MM in the masseter of mice (*n* = 5/group). Statistical comparison of the blotting data revealed that AChE **(H)** and CK-MM **(I)** expression in CRS mice was significantly higher than that in CON mice. **P < 0.01, ***P < 0.001.

**FIGURE 2:**
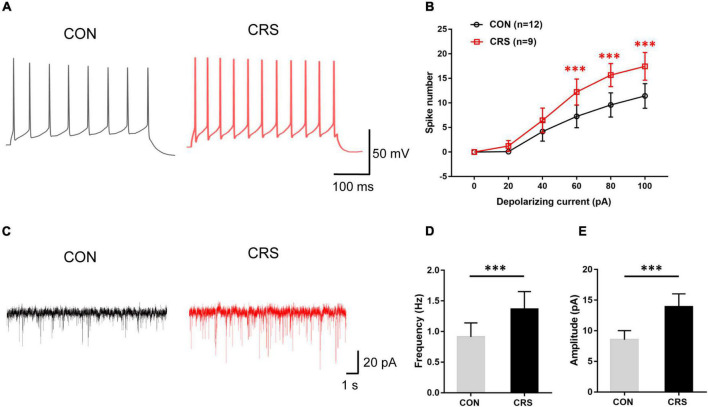
Effects of chronic restraint stress on action potential (AP) frequency and spontaneous excitatory postsynaptic currents (sEPSCs) of Vme neurons. **(A)** Representative traces evoked by intracellular injection of 80-pA depolarizing currents on Vme neurons from CON and CRS mice for 400 ms. **(B)** Data analysis showing that the firing numbers of APs from the CRS group were significantly increased compared with those from the CON group with injection of 60, 80, and 100 pA. **(C)** Representative traces of sEPSCs in Vme neurons recorded in the CON and CRS groups. Both frequency **(D)** and amplitude **(E)** were significantly higher in CRS mice than in CON mice. ***P < 0.001.

**FIGURE 3:**
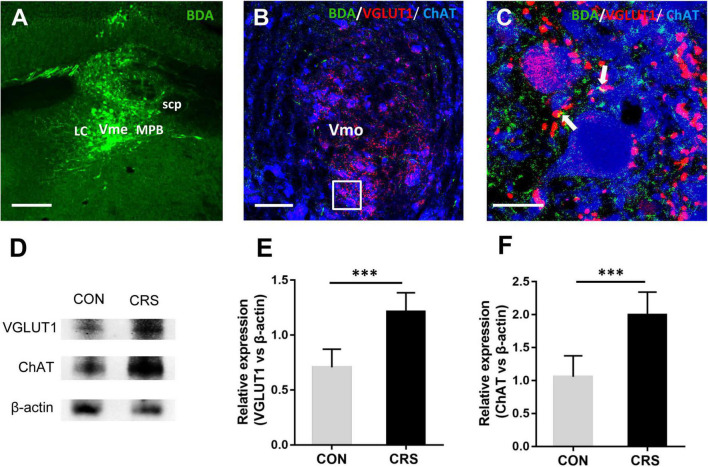
Neuronal tract tracing results of glutamatergic projections from the Vme to the Vmo and the expression of VGLUT1 and ChAT in the Vmo. **(A)** The injection site verified in the Vme. **(B)** Immunofluorescent triple labeling for BDA (green), VGLUT1 (red), and ChAT (blue) in the Vmo in mice injected with BDA into the Vme. The framed area in panel **(B)** is magnified in panel **(C)**. White arrows indicate axonal buttons labeled with both VGLUT1 (red) and BDA (green), which were merged (yellow) and in close apposition to ChAT-positive Vmo soma (blue). Scale bars: 200 μm in panel **(A)**; 100 μm in panel **(B)**; 20 μm in panel **(C)**. **(D)** Western blotting results of VGLUT1 and ChAT expression in the Vme of mice (*n* = 5/group). Statistical analysis revealed that VGLUT1 **(E)** and ChAT **(F)** expression in the CRS group was significantly higher than that in the CON group. ***P < 0.001.

**FIGURE 4:**
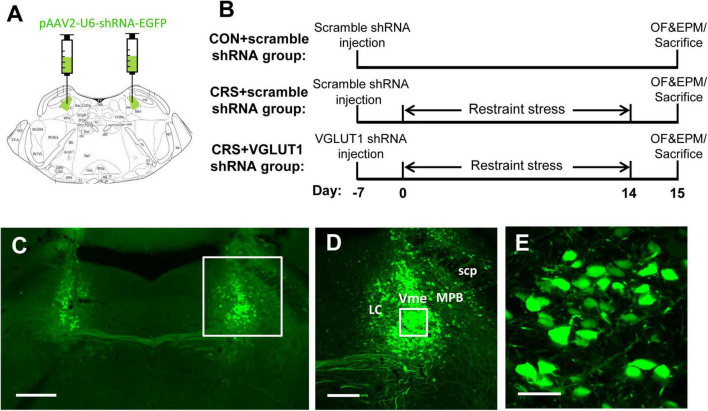
Representative schematic diagrams of the VGLUT1 shRNA vector injected into the bilateral Vme **(A)** and procedures for vector injection, restraint stress, behavioral tests, and sampling **(B)**. **(C–E)** Integration of virus vectors into Vme neurons. **(C)** Bilateral injection of VGLUT1 shRNA vectors into the Vme. The framed area in panel **(C)** and panel **(D)** is magnified in panel **(D)** and panel **(E)**, respectively. Successful transfection was morphologically verified by green fluorescent proteins knocked into the neurons. Scale bars: 500 μm in panel **(C)**; 200 μm in panel **(D)**; 50 μm in panel **(E)**.

### Statistical Analysis

All data are presented as the mean ± SD. Data from the first part except the spike number data in electrophysiology were analyzed by two-tailed unpaired Student’s *t*-test. Data from the third part except the spike number data in electrophysiology were analyzed by one-way ANOVA, followed by Tukey’s test for multiple comparisons. Data on the spike number in electrophysiology were tested with two-way ANOVA followed by Bonferroni’s *post hoc* test. All statistical analyses were performed using SPSS^®^ version 22.0 software (SPSS Inc., Chicago, IL, United States). *P* < 0.05 was considered statistically significant.

## Results

### Chronic Restraint Stress-Induced Anxiety-Like Behavior and Hyperactivity of the Masseter

Behavioral tests demonstrated that restraint stress led to noticeable anxiety-like behavior in mice. Figures 1A,B show the representative movement trials of mice from the CON and CRS groups in the OF and EPM tests. Compared to the mice in the CON group, the mice in the CRS group exhibited significantly decreased time spent in the center area (*P* < 0.01, Figure 1C) and a shorter moving distance in the center area (*P* < 0.001, Figure 1D) in the OF test. Additionally, a decreased percentage of OA retention time (*P* < 0.001, Figure 1E) and a decreased percentage of OA entries (*P* < 0.01, Figure 1F) were observed in the CRS group compared with the CON group in the EPM test. To evaluate the activities of masseter muscle after CRS, we observed the expression of AChE and CK-MM in the masseter muscle by Western blotting. The results showed that the expression levels of AChE and CK-MM in the CRS group were significantly increased compared with those in the CON group (*P* < 0.01, *P* < 0.01, Figures 1G–I), indicating masseter muscle hyperactivity under CRS conditions.

### Chronic Restraint Stress Caused Enhanced Excitability of Vme Neurons

Electrophysiological recording tests revealed significantly enhanced Vme neuronal activities in CRS mice. Figure 2A shows representative traces evoked by intracellular injection of 80-pA depolarizing currents in Vme neurons from CON and CRS mice by single-cell recording under the current clamp. Increasing spike numbers were elicited in CRS mice compared with CON mice when currents of 60, 80, and 100 pA (*P* < 0.001, *P* < 0.001, *P* < 0.001) were applied (Figure 2B). In voltage clamp mode, Figure 2C shows representative sEPSC traces at a −70-mV holding potential. The frequency and amplitude of sEPSCs in CRS mice were significantly elevated compared with those in CON mice (*P* < 0.001, *P* < 0.001, Figures 2D,E), indicating enhanced presynaptic and postsynaptic excitabilities. These results suggested that the activities of Vme neurons were obviously increased after CRS.

### Chronic Restraint Stress Linked to Masseter Hyperactivity by Glutamatergic Projections From the Vme to the Vmo

After anterograde tracer BDA injection into the Vme (Figure 3A), triple-labeling immunofluorescent staining was performed to detect whether projecting fibers and terminals from the Vme that formed closed contacts with Vmo neurons were glutamatergic. The results showed colocalization of BDA (green)- and VGLUT1 (red)-positive fibers and terminals in the ipsilateral Vmo (Figure 3B). Furthermore, by using ChAT to label Vmo neuronal somas, we observed a considerable amount of BDA and VGLUT1 double-labeled fibers and terminals that formed close contact with ChAT-positive somata (Figure 3C). These results suggested the existence of glutamatergic projections from the Vme to the Vmo. Furthermore, the Western blotting results showed that the expression levels of VGLUT1 and ChAT in Vmo were significantly increased after restraint stress for 2 weeks (*P* < 0.001, *P* < 0.001, Figures 3D–F) compared with those in CON mice.

### RNA Intervention in the Vme Failed to Attenuate the Anxiety-Like Behaviors Induced by Chronic Restraint Stress

To further confirm the involvement of Vme neurons in the masseter hyperactivity induced by CRS, we injected VGLUT1 shRNA into the bilateral Vme to intervene in glutamate expression (Figure 4A, 5.52 mm caudal to Bregma, Paxinos and Franklin, 2001). Behavioral changes in mice following restraint stress for 14 days were observed (Figure 4B). The validity of the AAV-vector infection was evaluated by EGFP expression in the injection site (Figure 4C). Higher-magnification images showed that the shRNA (EGFP, in green) had been successfully transfected into Vme neurons (Figures 4D,E).

**FIGURE 5:**
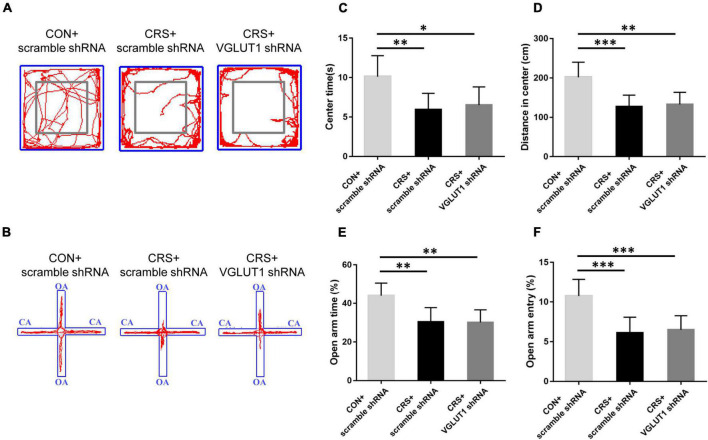
Effects of VGLUT1 shRNA vector injection on behavioral changes in mice. Representative movement trials of mice from the CON + scramble shRNA, CRS + scramble shRNA and CRS + VGLUT1 shRNA groups in the OF **(A)** and EPM **(B)** tests (*n* = 6/group). The gray square frames represent the center area in the OF. OA: open arm. CA: closed arm. Both CRS + scramble shRNA and CRS + VGLUT1 shRNA mice spent less time in the center area **(C)** and traveled a shorter distance in the center area **(D)** in the OF test than the CON + scramble shRNA mice and had lower percentages of time spent in the open arms **(E)** and entries into the open arms **(F)** in the EPM test than the CON + scramble shRNA mice. *P < 0.05, **P < 0.01, ***P < 0.001.

In Figure 5, representative movement trials from the CON + scramble shRNA, CRS + scramble shRNA, and CRS + VGLUT1 shRNA groups in the OF and EPM tests are shown. Compared to the mice in the CON + scramble shRNA group, the mice in both the CRS + scramble shRNA and CRS + VGLUT1 shRNA groups exhibited significantly decreased time spent in the center area (*P* < 0.01, CRS + scramble shRNA vs. CON + scramble shRNA, *P* < 0.05, CRS + VGLUT1 shRNA vs. CON + scramble shRNA, Figure 5C) and a shorter moving distance in the center area (*P* < 0.001, CRS + scramble shRNA vs. CON + scramble shRNA, *P* < 0.01, CRS + VGLUT1 shRNA vs. CON + scramble shRNA; Figure 5D) in the OF test. Furthermore, decreased percentages of OA retention time (*P* < 0.01, CRS + scramble shRNA vs. CON + scramble shRNA, *P* < 0.01, CRS + VGLUT1 shRNA vs. CON + scramble shRNA; Figure 5E) and OA entries (*P* < 0.001, CRS + scramble shRNA vs. CON + scramble shRNA, *P* < 0.001, CRS + VGLUT1 shRNA vs. CON + scramble shRNA; Figure 5F) were observed in both the CRS + scramble shRNA and CRS + VGLUT1 shRNA groups compared with the CON + scramble shRNA group. No statistical significance of the aforementioned parameters was found between CRS + scramble shRNA and CRS + VGLUT1 shRNA mice. The results of behavioral tests revealed that glutamatergic inhibition by RNA intervention in the Vme failed to alleviate the anxiety-like behaviors induced by CRS.

### RNA Intervention in the Vme Attenuated the Masseter Hyperactivity and Vme Neuron Excitability Induced by Chronic Restraint Stress

**FIGURE 6:**
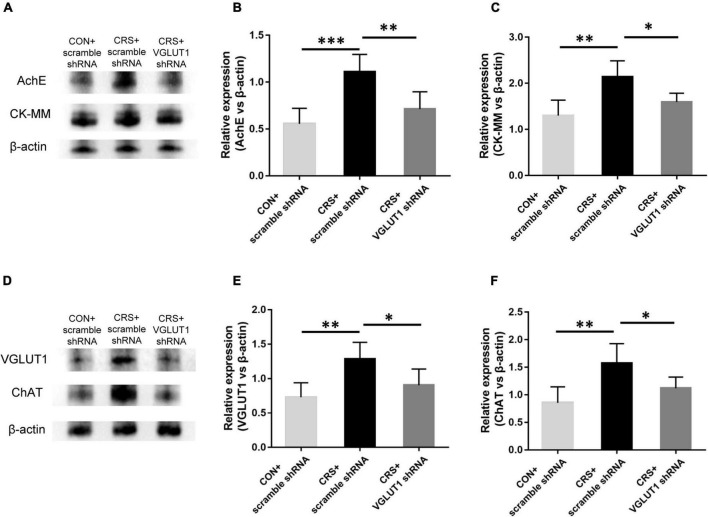
Effects of VGLUT1 shRNA vector injections on the expression of AChE and CK-MM in the masseter and the expression of VGLUT1 and ChAT in the Vmo (*n* = 5/group). **(A–C)** Western blotting analysis showed increased AChE and CK-MM protein levels in the masseters from CRS + scramble shRNA mice compared to CON + scramble shRNA mice. VGLUT1 shRNA vector injection significantly reversed AChE and CK-MM expression in the masseter compared to that in the UAC CRS + scramble shRNA group. **(D–F)** Western blotting analysis indicated that VGLUT1 and ChAT expression in the Vmo in the CRS + scramble shRNA group was significantly elevated compared to that in the CON + scramble shRNA group. VGLUT1 shRNA vector injection significantly reversed VGLUT1 and ChAT expression in the Vmo compared to that in the UAC CRS + scramble shRNA group. *P < 0.05, **P < 0.01, ***P < 0.001.

After behavioral tests, the mice from the CON + scramble shRNA, CRS + scramble shRNA, and CRS + VGLUT1 shRNA groups were sacrificed for Western blotting and electrophysiological recording measurements. Western blotting data indicated that AChE and CK-MM expression was significantly upregulated in the masseter muscles of CRS + scramble shRNA mice compared with CON + scramble shRNA mice (AChE, *P* < 0.001, CK-MM, *P* < 0.01, Figures 6A–C). VGLUT1 shRNA vector injection obviously downregulated AChE and CK-MM expression in the masseter muscles of CRS + VGLUT1 shRNA mice compared to CRS + scramble shRNA mice (AChE, *P* < 0.01, CK-MM, *P* < 0.05, Figures 6A–C). Consistently, significantly elevated VGLUT1 and ChAT expression was detected in the Vmo region in CRS + scramble shRNA mice compared with CON + scramble shRNA mice (VGLUT1, *P* < 0.01, ChAT, *P* < 0.01, Figures 6D–F). However, VGLUT1 shRNA rather than scramble shRNA injection into the Vme resulted in a significant decrease in VGLUT1 and ChAT expression in CRS mice (VGLUT1, *P* < 0.05, ChAT, *P* < 0.05, Figures 6D–F).

**FIGURE 7:**
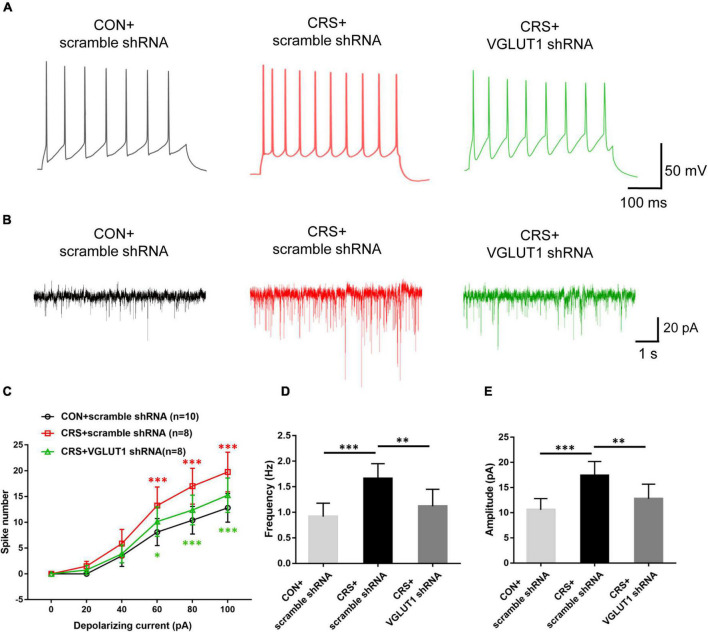
Effects of VGLUT1 shRNA vector injections on the action potential (AP) firing frequency and sEPSCs in Vme neurons in the CON + scramble shRNA, CRS + scramble shRNA and CRS + VGLUT1 shRNA groups. **(A)** Representative traces evoked by intracellular injection of 80-pA depolarizing currents on Vme neurons. **(B)** Representative traces of sEPSCs in Vme neurons. **(C)** The spike number was significantly increased in the CRS + scramble shRNA group compared to the CON + scramble shRNA group when currents of 60, 80, and 100 pA were applied. The shRNA injection group produced a significantly lower number of spikes in response to currents of 60, 80, and 100 pA compared with the CRS + scramble shRNA group. **(D,E)** Data analyses illustrated that the frequency and amplitude of sEPSCs in CRS + scramble shRNA mice were significantly higher than those in CON + scramble shRNA mice. The frequency and amplitude were significantly lower in the CRS + VGLUT1 shRNA mice than in the CRS + scramble shRNA mice. *P < 0.05, **P < 0.01, ***P < 0.001.

Electrophysiological recordings revealed that Vme neuronal activities were significantly enhanced in the CRS + scramble shRNA group. However, VGLUT1 shRNA injection reversed the enhanced neuronal activities of the Vme. Representative traces evoked by intracellular injection of 80-pA depolarizing currents in Vme neurons from the CON + scramble shRNA, CRS + scramble shRNA, and CRS + VGLUT1 shRNA groups under current clamp are shown in Figure 7A. In the CRS + scramble shRNA group, increasing elicited spike numbers were observed compared with those in the CON + scramble shRNA group at currents of 60, 80, and 100 pA (*P* < 0.001, *P* < 0.001, *P* < 0.001, Figure 7C). However, VGLUT1 shRNA injection produced reduced spike numbers compared with those in the CRS + scramble shRNA group in response to currents at 60, 80, and 100 pA (*P* < 0.05, *P* < 0.001, *P* < 0.001, Figure 7C). In parallel, in voltage clamp mode, Figure 7B shows representative sEPSC traces at a −70-mV holding potential. The frequency and amplitude of Vme neuron sEPSCs in CRS + scramble shRNA mice were significantly higher than those in CON + scramble shRNA mice (*P* < 0.001, *P* < 0.001, Figures 7D,E). However, VGLUT1 shRNA injection decreased both the frequency and amplitude of sEPSCs in Vme neurons (*P* < 0.01, *P* < 0.01, Figures 7D,E). These data suggested that the activities of Vme neurons were overexcited under CRS conditions and could be reversed by Vme glutamatergic RNA intervention.

## Discussion

Previous studies have demonstrated a strong correlation between psychological stress and masticatory muscle disorder, which is the main reported symptom of TMD patients (Owczarek et al., 2020; Yap and Natu, 2020; Rofaeel et al., 2021). Dentists are interested in determining the central mechanisms underlying this phenomenon. In the present study, by establishing an animal model of CRS, we observed obviously increased expression of AChE and CK-MM, indicating overactivity in the masseter muscle. Moreover, we identified the involvement of the Vme, a key nucleus in orosensory–motor control, in the process of stress-induced masseter overactivity. Electrophysiological recordings showed that Vme neurons exhibited enhanced excitability after CRS. Then, an increase in glutamatergic neurotransmission from the Vme to the Vmo was revealed by Western blotting measurement of VGLUT1 overexpression in the Vmo. Furthermore, injection of VGLUT1 shRNA into the Vme could significantly suppress the enhanced overexcitability in the Vme, downregulate the overexpression of VGLUT1 and ChAT in the Vmo, and thus attenuate the elevated overactivities of the masseter.

In the current study, we employed an animal model of CRS to mimic the stress condition in humans and investigated behavioral changes. Restraint stress, which can cause physiological and psychological discomfort by restricting an animal’s free movement to a moderate extent, is widely used in studies designed to investigate negative emotions such as anxiety or depression (Chiba et al., 2012; Huo et al., 2017; Liu et al., 2020). The OF and EPM tests were used to evaluate the negative emotions in mice (Wang et al., 2015; Segklia et al., 2019). Significantly decreased time spent and distances traveled in the center area in the OF test and reduced time and entries into OAs in the EPM demonstrated that an obvious anxious emotion had appeared in mice after CRS, which is in accordance with previous studies showing that 1–3 weeks of restraint stress can elicit anxiety-like behavior (Chiba et al., 2012; Liu et al., 2020).

To evaluate the change in masticatory muscle function of mice under CRS, we chose AChE and CK-MM as the main predictors of masseter activity. AChE is one of the main functional proteins of the neuromuscular junction and is responsible for the fast hydrolysis of acetylcholine, which is the key neurotransmitter for skeletal muscle contraction (Pregelj et al., 2007; Bonansea et al., 2016; Wall and Woolley, 2020). Moreover, CK-MM is the most commonly distributed CK isoenzyme in skeletal muscle (Hornemann et al., 2003). The elevated level of CK-MM often indicates overactivity of muscle and resulting cell injury (Brancaccio et al., 2007; Miranda-Vilela et al., 2012). The Western blotting results revealed significantly increased expression of AChE and CK-MM in masseter tissue, suggesting that CRS may cause enhanced masticatory muscle activity, which may consequently lead to muscle dysfunction and thus trigger or aggravate the symptoms of TMD. Nevertheless, in the central nervous system, the mechanisms of how stress-induced reactions convert into hyperactivities of jaw muscles remain unclear.

In the midbrain, the Vme is a central relay region for transmitting proprioceptive input from the orofacial area and in turn projecting efferents into the oromotor nucleus, coordinating orofacial movements, including chewing and swallowing (Lazarov, 2002; Fortin et al., 2021). In the process of mastication the jaw muscle spindles are simulated by changed muscle length. The primary afferent neurons in the Vme are thus activated by peripheral endings located in these jaw muscle spindles. Through the central projection to the Vmo, oromotor neurons are also activated, and muscle contraction is elicited (Lazarov, 2007; Zhang et al., 2012; Lipovsek et al., 2017). Unlike other primary afferent sensory neurons, such as trigeminal ganglion neurons, the Vme is a unique nucleus containing primary afferent sensory neurons that situate their somata in the brain. Due to their ectopic location within the brain, mesencephalic trigeminal neurons receive synaptic inputs from various areas in the whole brain, which may potentially modify their output to the Vmo (Lipovsek et al., 2017; Fortin et al., 2021). Thus, we focused on whether excitability changes occurred in Vme neurons as masseter overactivity was initiated by CRS.

In the present study, we demonstrated that CRS could induce marked neuronal excitability enhancement in the Vme, and injection of VGLUT1 shRNA into the Vme significantly reversed this overexcitability. In previous studies, Vme neurons have been demonstrated to be glutamatergic and to utilize VGLUT1 as the carrier for synaptic neurotransmission to Vmo (Pang et al., 2006; Park et al., 2018). In addition, most of the central axons of Vme neurons project to ipsilateral Vmo motor neurons (Pang et al., 2006, 2009), which directly innervate jaw muscle movement. Hence, to some extent, the expression level of VGLUT1 in the Vmo may reflect changes in glutamatergic projections from the Vme. We next detected the expression of ChAT, which is a typical neural marker for motor neurons in the Vmo (Salvaterra, 1987; Lee et al., 2019). The Western blotting results indicated obviously increased levels of VGLUT1 and ChAT expression caused by CRS but significantly decreased VGLUT1 and ChAT levels after VGLUT1 shRNA interference in the Vme. Furthermore, the motor activity level of the masseter was also inhibited with significantly reduced AChE and CK-MM expression by VGLUT1 shRNA injection into the Vme. As for the inhibited Vme neurons overexcitability induced by VGLUT1 shRNA interference in the Vme, the possible reasons might be that the changed activity of masseter muscle may alter the proprioceptive afferent projections to Vme, resulting in the changed activity of Vme neurons. Taken together, these results suggested that excitation of Vme neurons is involved in the induction of masseter overactivity by CRS, which might work through the intensified glutamatergic projection of the Vme-Vmo pathway.

To date, literature regarding the central mechanisms of stress-induced masticatory muscle dysfunction is limited. The Vme, which is described as a displaced sensory ganglion (Lazarov, 2007; Lipovsek et al., 2017; Fortin et al., 2021), has an inherent anatomically ambiguous nature. On one hand, it plays a vital role in generating and regulating mastication movement; on the other hand, it also receives large amounts of central projections owing to its central situation, which is different from other primary sensory ganglions. Perhaps this particular feature of Vme may explain our results showing that activation of Vme neurons may contribute to the masseter overactivity induced by stress. However, in this study, VGLUT1 shRNA interference in the Vme failed to alleviate stress-induced anxiety-like behaviors in the OF and EPM tests, suggesting that the Vme might be one of relaying regions through which stress can exert an effect on masseter activity. Consequently, we speculate that other higher brain centers instead of the Vme are presumably involved in the induction and regulation of anxious emotions in CRS mice. The central nucleus of the amygdala (CeA), which plays an important role in negative emotional perception and regulation, has been reported to send direct projections to the Vme (Lazarov et al., 2011; Shirasu et al., 2011). The CeA may thus be a key region responsible for such anxious behaviors in CRS mice. Nonetheless, further studies are still needed to explore the possible neural pathways, such as the CeA-Vme involved in CRS-induced negative emotions. In addition, as the masseter was the only masticatory muscle selected for evaluation in the present study, the changes of other muscles of mastication in stressed condition may also be included in the further studies.

In summary, the present study indicated that CRS can excite Vme neurons, lead to enhanced glutamatergic excitatory projections from the Vme to the Vmo, and result in overactivity of the masseter muscle. These findings provide a novel central mechanism underlying the correlation between psychological factors and TMD.

## Data Availability Statement

The raw data supporting the conclusions of this article will be made available by the authors, without undue reservation.

## Ethics Statement

The animal study was reviewed and approved by the Animal Use and Care Committee for Research and Education of the Fourth Military Medical University.

## Author Contributions

Y-JZ, YL, and JW established the animal model and tract tracing experiments. YL, QL, and RL conducted the behavioral tests. Y-JZ, YL, and TT performed the Western blotting assays. Y-JZ, YL, JW, and Z-MZ carried out the immunostaining experiments. Y-JZ and JW performed the electrophysiology study. Y-JZ, YL, and JW wrote the manuscript. QL, MZ, and Y-JC revised the manuscript. MZ and Y-JC conceived the project and coordinated and supervised the experiments. All authors discussed the manuscript and read and approved the final manuscript.

## Conflict of Interest

The authors declare that the research was conducted in the absence of any commercial or financial relationships that could be construed as a potential conflict of interest.

## Publisher’s Note

All claims expressed in this article are solely those of the authors and do not necessarily represent those of their affiliated organizations, or those of the publisher, the editors and the reviewers. Any product that may be evaluated in this article, or claim that may be made by its manufacturer, is not guaranteed or endorsed by the publisher.

## References

[B1] Abe C. Inoue T. Inglis M. A. Viar K. E. Huang L. Ye H. (2017). C1 neurons mediate a stress-induced anti-inflammatory reflex in mice. *Nat. Neurosci.* 20 700–707. 10.1038/nn.4526 28288124 PMC5404944

[B2] Al-Khotani A. Naimi-Akbar A. Gjelset M. Albadawi E. Bello L. Hedenberg-Magnusson B. (2016). The associations between psychosocial aspects and TMD-pain related aspects in children and adolescents. *J. Headache Pain* 17:30. 10.1186/s10194-016-0622-0 27044436 PMC4820412

[B3] Almukhtar R. M. Fabi S. G. (2019). The masseter muscle and its role in facial contouring, aging, and quality of life: a literature review. *Plast. Reconstr. Surg.* 143 39e–48e. 10.1097/prs.0000000000005083 30303926

[B4] Benoliel R. Svensson P. Heir G. M. Sirois D. Zakrzewska J. Oke-Nwosu J. (2011). Persistent orofacial muscle pain. *Oral Dis.* 17 23–41. 10.1111/j.1601-0825.2011.01790.x 21382137

[B5] Bonansea R. I. Wunderlin D. A. Amé M. V. (2016). Behavioral swimming effects and acetylcholinesterase activity changes in Jenynsia multidentata exposed to chlorpyrifos and cypermethrin individually and in mixtures. *Ecotoxicol. Environ. Saf.* 129 311–319. 10.1016/j.ecoenv.2016.03.043 27060258

[B6] Brancaccio P. Maffulli N. Limongelli F. M. (2007). Creatine kinase monitoring in sport medicine. *Br. Med. Bull.* 81 209–230. 10.1093/bmb/ldm014 17569697

[B7] Chiba S. Numakawa T. Ninomiya M. Richards M. C. Wakabayashi C. Kunugi H. (2012). Chronic restraint stress causes anxiety- and depression-like behaviors, downregulates glucocorticoid receptor expression, and attenuates glutamate release induced by brain-derived neurotrophic factor in the prefrontal cortex. *Prog. Neuropsychopharmacol. Biol. Psychiatry* 39 112–119. 10.1016/j.pnpbp.2012.05.018 22664354

[B8] Costa Y. M. Ariji Y. Ferreira D. Bonjardim L. R. Conti P. C. R. Ariji E. (2018). Muscle hardness and masticatory myofascial pain: assessment and clinical relevance. *J. Oral Rehabil.* 45 640–646. 10.1111/joor.12644 29745983

[B9] Dahlström L. Carlsson G. E. (2010). Temporomandibular disorders and oral health-related quality of life. A systematic review. *Acta Odontol. Scand.* 68 80–85. 10.3109/00016350903431118 20141363

[B10] Fillingim R. B. Ohrbach R. Greenspan J. D. Knott C. Diatchenko L. Dubner R. (2013). Psychological factors associated with development of TMD: the OPPERA prospective cohort study. *J. Pain* 14 T75–T90. 10.1016/j.jpain.2013.06.009 24275225 PMC3855656

[B11] Fortin S. M. Chen J. Grill H. J. Hayes M. R. (2021). The mesencephalic trigeminal nucleus controls food intake and body weight *via* hindbrain POMC projections. *Nutrients* 13:1642. 10.3390/nu13051642 34068091 PMC8152732

[B12] Gameiro G. H. Da Silva, Andrade A. Nouer D. F. Ferraz de Arruda Veiga M. C. (2006). How may stressful experiences contribute to the development of temporomandibular disorders? *Clin. Oral Investig.* 10 261–268. 10.1007/s00784-006-0064-1 16924558

[B13] Hornemann T. Kempa S. Himmel M. Hayess K. Fürst D. O. Wallimann T. (2003). Muscle-type creatine kinase interacts with central domains of the M-band proteins myomesin and M-protein. *J. Mol. Biol.* 332 877–887. 10.1016/s0022-2836(03)00921-5 12972258

[B14] Huo R. Zeng B. Zeng L. Cheng K. Li B. Luo Y. (2017). Microbiota modulate anxiety-like behavior and endocrine abnormalities in hypothalamic-pituitary-adrenal axis. *Front. Cell. Infect. Microbiol.* 7:489. 10.3389/fcimb.2017.00489 29250490 PMC5715198

[B15] Kindler S. Samietz S. Houshmand M. Grabe H. J. Bernhardt O. Biffar R. (2012). Depressive and anxiety symptoms as risk factors for temporomandibular joint pain: a prospective cohort study in the general population. *J. Pain* 13 1188–1197. 10.1016/j.jpain.2012.09.004 23141187

[B16] Kitagawa K. Kodama N. Manda Y. Mori K. Furutera H. Minagi S. (2021). Effect of masseter muscle activity during wakefulness and sleep on tooth wear. [Epub ahead of print]. 10.2186/jpr.JPR_D_21_00171 34955483

[B17] Lazarov N. E. (2002). Comparative analysis of the chemical neuroanatomy of the mammalian trigeminal ganglion and mesencephalic trigeminal nucleus. *Prog. Neurobiol.* 66 19–59. 10.1016/s0301-0082(01)00021-1 11897404

[B18] Lazarov N. E. (2007). Neurobiology of orofacial proprioception. *Brain Res. Rev.* 56 362–383. 10.1016/j.brainresrev.2007.08.009 17915334

[B19] Lazarov N. E. Usunoff K. G. Schmitt O. Itzev D. E. Rolfs A. Wree A. (2011). Amygdalotrigeminal projection in the rat: an anterograde tracing study. *Ann. Anat.* 193 118–126. 10.1016/j.aanat.2010.12.004 21333509

[B20] Lee N. Wanek H. A. MacLennan A. J. (2019). Muscle ciliary neurotrophic factor receptor α helps maintain choline acetyltransferase levels in denervated motor neurons following peripheral nerve lesion. *Exp. Neurol.* 317 202–205. 10.1016/j.expneurol.2019.03.009 30902524 PMC6544478

[B21] Lin W. Zhao Y. Cheng B. Zhao H. Miao L. Li Q. (2019). NMDAR and JNK activation in the spinal trigeminal nucleus caudalis contributes to masseter hyperalgesia induced by stress. *Front. Cell. Neurosci.* 13:495. 10.3389/fncel.2019.00495 31798413 PMC6868050

[B22] Lipovsek M. Ledderose J. Butts T. Lafont T. Kiecker C. Wizenmann A. (2017). The emergence of mesencephalic trigeminal neurons. *Neural Dev.* 12:11. 10.1186/s13064-017-0088-z 28637511 PMC5480199

[B23] Liu W. Z. Zhang W. H. Zheng Z. H. Zou J. X. Liu X. X. Huang S. H. (2020). Identification of a prefrontal cortex-to-amygdala pathway for chronic stress-induced anxiety. *Nat. Commun.* 11:2221. 10.1038/s41467-020-15920-7 32376858 PMC7203160

[B24] Liu X. Zhou K. X. Yin N. N. Zhang C. K. Shi M. H. Zhang H. Y. (2019). Malocclusion generates anxiety-like behavior through a putative lateral habenula-mesencephalic trigeminal nucleus pathway. *Front. Mol. Neurosci.* 12:174. 10.3389/fnmol.2019.00174 31427925 PMC6689965

[B25] Miranda-Vilela A. L. Akimoto A. K. Lordelo G. S. Pereira L. C. Grisolia C. K. Klautau-Guimarães M. D. N. (2012). Creatine kinase MM TaqI and methylenetetrahydrofolate reductase C677T and A1298C gene polymorphisms influence exercise-induced C-reactive protein levels. *Eur. J. Appl. Physiol.* 112 941–950. 10.1007/s00421-011-1961-9 21706313

[B26] Nishioka G. J. Montgomery M. T. (1988). Masticatory muscle hyperactivity in temporomandibular disorders: is it an extrapyramidally expressed disorder? *J. Am. Dent. Assoc.* 116 514–520. 10.14219/jada.archive.1988.0320 2897984

[B27] Nitecka-Buchta A. Walczynska-Dragon K. Kempa W. M. Baron S. (2019). Platelet-Rich Plasma Intramuscular Injections - Antinociceptive Therapy in Myofascial Pain Within Masseter Muscles in Temporomandibular Disorders Patients: a Pilot Study. *Front. Neurol.* 10:250. 10.3389/fneur.2019.00250 30941095 PMC6433706

[B28] Oghli I. List T. Su N. Häggman-Henrikson B. (2020). The impact of oro-facial pain conditions on oral health-related quality of life: a systematic review. *J. Oral Rehabil.* 47 1052–1064. 10.1111/joor.12994 32415993

[B29] Osiewicz M. A. Lobbezoo F. Loster B. W. Loster J. E. Manfredini D. (2018). Frequency of temporomandibular disorders diagnoses based on RDC/TMD in a Polish patient population. *Cranio* 36 304–310. 10.1080/08869634.2017.1361052 28792365

[B30] Owczarek J. E. Lion K. M. Radwan-Oczko M. (2020). Manifestation of stress and anxiety in the stomatognathic system of undergraduate dentistry students. *J. Int. Med. Res.* 48:300060519889487. 10.1177/0300060519889487 32046557 PMC7105728

[B31] Pang Y. W. Ge S. N. Nakamura K. C. Li J. L. Xiong K. H. Kaneko T. (2009). Axon terminals expressing vesicular glutamate transporter VGLUT1 or VGLUT2 within the trigeminal motor nucleus of the rat: origins and distribution patterns. *J. Comp. Neurol.* 512 595–612. 10.1002/cne.21894 19058187

[B32] Pang Y. W. Li J. L. Nakamura K. Wu S. Kaneko T. Mizuno N. (2006). Expression of vesicular glutamate transporter 1 immunoreactivity in peripheral and central endings of trigeminal mesencephalic nucleus neurons in the rat. *J. Comp. Neurol.* 498 129–141. 10.1002/cne.21047 16856164

[B33] Park S. K. Ko S. J. Paik S. K. Rah J. C. Lee K. J. Bae Y. C. (2018). Vesicular glutamate transporter 1 (VGLUT1)- and VGLUT2-immunopositive axon terminals on the rat jaw-closing and jaw-opening motoneurons. *Brain Struct. Funct.* 223 2323–2334. 10.1007/s00429-018-1636-y 29476240

[B34] Paxinos G. Franklin K. (2001). *The Mouse Brain in Stereotaxic Coordinates.* Cambridge: Academic Press.

[B35] Pregelj P. Trinkaus M. Zupan D. Trontelj J. J. Sketelj J. (2007). The role of muscle activation pattern and calcineurin in acetylcholinesterase regulation in rat skeletal muscles. *J. Neurosci.* 27 1106–1113. 10.1523/jneurosci.4182-06.2007 17267565 PMC6673202

[B36] Rofaeel M. Chow J. C. Cioffi I. (2021). The intensity of awake bruxism episodes is increased in individuals with high trait anxiety. *Clin. Oral Investig.* 25 3197–3206. 10.1007/s00784-020-03650-5 33098032

[B37] Rosales V. P. Ikeda K. Hizaki K. Naruo T. Nozoe S. Ito G. (2002). Emotional stress and brux-like activity of the masseter muscle in rats. *Eur. J. Orthod.* 24 107–117. 10.1093/ejo/24.1.107 11887374

[B38] Salvaterra P. M. (1987). Molecular biology and neurobiology of choline acetyltransferase. *Mol. Neurobiol.* 1 247–280. 10.1007/bf02936610 3077061

[B39] Segklia K. Stamatakis A. Stylianopoulou F. Lavdas A. A. Matsas R. (2019). Increased anxiety-related behavior, impaired cognitive function and cellular alterations in the brain of Cend1-deficient mice. *Front. Cell. Neurosci.* 12:497. 10.3389/fncel.2018.00497 30760981 PMC6361865

[B40] Shah N. Melo L. Reid W. D. Cioffi I. (2019). Masseter Deoxygenation in Adults at Risk for Temporomandibular Disorders. *J. Dent. Res.* 98 666–672. 10.1177/0022034519837249 30946624

[B41] Shirasu M. Takahashi T. Yamamoto T. Itoh K. Sato S. Nakamura H. (2011). Direct projections from the central amygdaloid nucleus to the mesencephalic trigeminal nucleus in rats. *Brain Res.* 1400 19–30. 10.1016/j.brainres.2011.05.026 21640334

[B42] Silman I. Sussman J. L. (2008). Acetylcholinesterase: how is structure related to function? *Chem. Biol. Interact.* 175 3–10. 10.1016/j.cbi.2008.05.035 18586019

[B43] Simoen L. Van den Berghe L. Jacquet W. Marks L. (2020). Depression and anxiety levels in patients with temporomandibular disorders: comparison with the general population. *Clin. Oral Investig.* 24 3939–3945. 10.1007/s00784-020-03260-1 32219566

[B44] Slade G. D. Ohrbach R. Greenspan J. D. Fillingim R. B. Bair E. Sanders A. E. (2016). Painful temporomandibular disorder: decade of discovery from OPPERA studies. *J. Dent. Res.* 95 1084–1092. 10.1177/0022034516653743 27339423 PMC5004239

[B45] Song F. Li Q. Wan Z. Y. Zhao Y. J. Huang F. Yang Q. (2014). Lamotrigine reverses masseter overactivity caused by stress maybe *via* Glu suppression. *Physiol. Behav.* 137 25–32. 10.1016/j.physbeh.2014.06.017 24955497

[B46] Wall E. M. Woolley S. C. (2020). Acetylcholine in action. *Elife* 9:e57515. 10.7554/eLife.57515 32425156 PMC7237203

[B47] Wang J. Li Z. H. Feng B. Zhang T. Zhang H. Li H. (2015). Corticotrigeminal projections from the insular cortex to the trigeminal caudal subnucleus regulate orofacial pain after nerve injury *via* extracellular signal-regulated kinase activation in insular cortex neurons. *Front. Cell. Neurosci.* 9:493. 10.3389/fncel.2015.00493 26733817 PMC4689789

[B48] Yap A. U. Natu V. P. (2020). Inter-relationships between pain-related temporomandibular disorders, somatic and psychological symptoms in Asian youths. *J. Oral Rehabil.* 47 1077–1083. 10.1111/joor.13033 32515495

[B49] Zhang J. Luo P. Ro J. Y. Xiong H. (2012). Jaw muscle spindle afferents coordinate multiple orofacial motoneurons *via* common premotor neurons in rats: an electrophysiological and anatomical study. *Brain Res.* 1489 37–47. 10.1016/j.brainres.2012.10.021 23085474

[B50] Zimmermann M. (1983). Ethical guidelines for investigations of experimental pain in conscious animals. *Pain* 16 109–110. 10.1016/0304-3959(83)90201-4 6877845

